# Influences of azolla-derived biofertilizer and compost on soil chemical properties and rice growth in contaminated soil

**DOI:** 10.1371/journal.pone.0344652

**Published:** 2026-03-16

**Authors:** Talaat R. El-beshbeshy, Asmaa Z. El Kady, Rania M. El Shal, Nazih Y. Rebouh, Mohamed S. Shokr, Naglaa E. Khalafallah

**Affiliations:** 1 Soil and Water Department, Faculty of Agriculture, Tanta University, Tanta, Egypt; 2 Department of Environmental Management, Institute of Environmental Engineering RUDN University, Moscow, Russia; Universiti Brunei Darussalam, BRUNEI DARUSSALAM

## Abstract

Pollution caused by heavy metals and extensive use of synthetic fertilizers has harmful effects on soil organic matter content, soil health, environmental safety, and human health. To overcome these problems, using several types of amendments to improve soil conditions can boost crop productivity. It has been proposed to use Azolla as compost and a biofertilizer to enhance soil quality and promote rice growth in contaminated soil. This study was carried out at the Department of Soil and Water, Faculty of Agriculture, Tanta University, Egypt, during summer season of 2021. To investigate the effect of Azolla in two forms: as a green manure (*Azolla pinnata*) and as compost under different levels of nitrogen fertilizer on the growth of the rice crop (Sakha 104). In addition, the study investigated some soil chemical properties contaminated by heavy metals such as Cu, Pb, and Zn. To achieve the aim of this study, soil samples from two different sites were collected from Kafr El-Zayat, Gharbia Governorate, Egypt. Thirteen treatments were used to fulfill the study's objectives. The results showed that the application of *Azolla pinnata* and Azolla compost significantly improved the grain production of rice, increased cation exchange capacity (CEC), % of organic matter (OM), available and total NPK, and prevented the pH from rising. The total and available levels of Zn and Cu in our samples dropped to an acceptable level, leading to the adoption of remedial measures according to Canadian soil quality recommendations, although the total Pb content remained high. In the end, our research advances important Sustainable Development Goals (SDGs) of the United Nations, such as Life on Land (SDG 15) by restoring soil health, Responsible Consumption and Production (SDG 12) by encouraging circular bioeconomies, and Zero Hunger (SDG 2) and Good Health (SDG 3) through safer food production.

## 1. Introduction

Fifty percent of the world's population depends on rice (*Oryza sativa* L.) for sustenance, making it the second most important cereal crop after wheat [1]. In 2018, there were approximately 164.19 million hectares of rice cultivated worldwide, with an annual grain production of 756.74 million tons and 4.60 tons of grains per hectare [2]. Producing high grain yields to meet the food needs of constantly expanding populations, millions of tons of synthetic fertilizers are required to cultivate such vast areas [3,4]. Rice is one of the most significant food and export crops in Egypt, occupying most of the Delta's agricultural land throughout the summer [5]. Egypt is the largest producer of rice in both Africa and the Middle East. Farmers employ a variety of pesticides to protect their crops in response to rising population growth and consequent demand for rice; these pesticides use may affect heavy metals concentrations in soils [6,7]. Increased uptake of heavy metals by vegetables and cereal crops due to elevated soil concentrations of heavy metals may pose a risk to public health through food chains [8,9]. In addition, the variety of plant species in an agroecosystem may decline as a result of widespread and continuous pesticide use [10,11]. Environmental contaminants include heavy metals and metalloids (HMs) [12]. They are considered pollutants of agricultural soil, as high concentrations of HMs can have a detrimental effect on crop yield and health [13,14].

The toxicity of heavy metals, their non-biodegradable nature, their tendency to accumulate, and their multiple sources make contamination and accumulation major environmental concerns [15]. According to Karaca et al., [16], the main metals responsible for soil contamination include include copper (Cu), cadmium (Cd), nickel (Ni), chromium (Cr), zinc (Zn), and lead (Pb). These heavy metals enter the soil from multiple sources, including sewage sludge, farmyard manure, rapid industrialization, atmospheric deposition, and the prolonged use of synthetic fertilizers [17,18]. For growth and maintenance, plants need specific heavy metals, and excessive amounts can be harmful to plants. The mechanisms that allow the accumulation of essential metals also facilitate the uptake of non-essential metals [19]. Heavy metals and trace elements differ primarily in their physiological role and toxicity. Trace elements (e.g., Zn, Cu, Mn, Fe) are essential micronutrients, required at low concentrations for plant growth and for metabolic processes such as photosynthesis and enzyme activation. In contrast, heavy metals (e.g., Cd, Pb, Hg) are generally non-essential, non-biodegradable, and toxic even at trace amounts; they can damage DNA, inhibit enzymes, and induce oxidative stress, providing no beneficial role in plant metabolism. Some trace elements are classified as heavy metals (e.g., Cu, Zn, Mn, Fe).These are essential for at low concentrations but become toxic at high levels. Excessive levels of all heavy metals disrupt photosynthesis, reduce root and shoot elongation, inhibit germination, and cause oxidative stress, thereby hindering overall plant growth. Metals negatively impact plants directly and indirectly when their concentrations exceed levels because of their non-biodegradable nature. Direct harmful effects include inhibition of the cytoplasmic enzymes and oxidative stress-induced cell structural destruction [20,21]. The displacement of nutrients at plant cation exchange sites is an example of an indirect harmful effect [22]. Heavy metals affect the growth, morphology, and metabolism of soil microorganisms through functional disturbance, protein denaturation, or the destruction and cell membrane disruption [23]. This negative impact on microbial population size and activity can indirectly impair plant growth. For example, a reduction in organic matter decomposition caused by a high metal level reduces the population of beneficial soil microorganisms, leading to a loss of soil nutrients. Heavy metals also interfere with the soil microbial activity, inhibiting enzymes essential for plant metabolism. Both direct and indirect toxic effects cause a decrease in plant growth, which can occasionally lead to plant death [24].

Because enzyme activity is crucial for vital metabolic functions, heavy metals inhibit growth by disturbing this activity [25]. According to Siedlecka and Krupa [26], heavy metals can bind firmly to oxygen, nitrogen, and sulfur atoms, in addition to sulfhydryl groups and disulfide bonds. This can damage protein secondary structures and interfere with enzyme function, thereby disrupting a variety of metabolic pathways. The metabolic activities of plants, include the conversion of nutrients and the breakdown of organic substances into forms that can be used. These cycles and the availability can become unbalanced when excessive concentrations of heavy metals are present. Consequently, the nutrient quality declines, reducing overall soil quality and impairing plant growth and development [27]. Extensive research has been conducted to address soil pollution by developing practical methods for risk assessment and remediation [28]. To address this issue, researchers have investigated both non-biological and biological approaches. Many techniques are available, such as chemical, biological, and physical techniques [29]. The mechanism and efficiency of phytoremediation depend on the contaminant type and soil characteristics [30]*,* as each mechanism differentially affects the volume, mobility, and toxicity of contaminants [31,32]**.** Plants that accumulate high concentrations of metals are called hyperaccumulators and can accumulate 50–100 times more metal than non- accumulator plants [33]. Azolla (*Azolla filiculoides*) is a small aquatic fern that grows in lakes, pools, and marshes with calm water. It can fix nitrogen from the atmosphere by forming a symbiotic relationship with cyanobacteria, such as *Anabaena azollae*, which are found in the dorsal lobes of its leaves. Azolla’s high nitrogen content makes it valuable as a green manure and feedstock for increasing crop yield [34–36]. Due to its favorable effect on reducing global warming and methane emissions, azolla compost is considered an environmentally sustainable agricultural practice [37]. It also serves as a cost-effective, natural, and efficient phytoremediation method for removing heavy metals from soil; by reducing soil heavy metal concentration, it limits subsequent uptake by plants. Azolla has a lot of benefits, characterization such as: 1) its being fast- growing, free-floating freshwater fern; 2) its rapid biomass doubling time of 3–5 days; and 3) its ability to fix atmospheric nitrogen by forming a symbiotic association with the blue green algae, *A. azollae* [38].

In the Nile Delta of Egypt like El-Gharbia Governorate, the excessive use of sewage sludge, extensive, fertilizers and manure causes hazardous effects on plants, animals, and human health. Additionally, the discharge of fluid and solid wastes with different contaminants into the environment results groundwater degradation [39,40]. Furthermore, the Kafr El-Zayat industrial area discharges liquid waste from the factories producing superphosphate, sulfur compounds, oil and pesticides [41]. Thus, the primary objective of this study is to examine the effects of two forms of azolla green (*Azolla pinnata*)—fresh biomass as a biofertilizer and azolla compost as a soil amendment — under different nitrogen fertilizer levels on the growth of rice (Sakha 104) and on selected physiochemical properties in Cu, Pb, and Zn-contaminated soil in the Nile Delta, Egypt. The novelty of this study lies in demonstrating the potential of Azolla for phytoremediation, given its rapid uptake and tolerance of various heavy metals, including Cu, Pb, and Zn. Its high biomass production, ease of harvest, and nitrogen-fixing capacity make it a sustainable, cost-effective, and environmentally sound method for remediating contaminated soils and water.

## 2. Materials and methods

The research work was conducted during the summer of 2021 at the Department of Soil and water, Faculty of Agriculture, Tanta University. A Pot experiment was designed to investigate the effects of green azolla (*Azolla pinnata*) as a biofertilizer, azolla compost and their interaction on improving soil properties and growth of rice under contaminated soil. The specific objectives were to: i) assess the individual and combined effects of *A. pinnata* and its compost on soil properties and plant growth, ii) evaluate the phytoremediation potential of *A. pinnata* for extracting heavy metals from the contaminated soil, iii) compare the effectivenss of *A. pinnata* biomass versus azolla compost as a soil amendments for heavy metals remediation Composite soil samples from (0–30 cm depth) were collected. Ten surface samples (each from a of 1m^2^ area) were taken from the study area in Kafr El Zayat Center. These were thoroughly mixed in the field (175 m^2^) to form a homogeneous composite, from which 1000 kg of soil was obtained for the pot experiment. The properties of the experimental soil are presented in Table 1.

**Table 1:** Some characteristics of the soil used in the study.

Properties	Unit	Soil
pH soil water suspension 1:2.5 (w/v)		8.14
Electro conductivity (1:5 soil-water extract)	dS m^-1^	0.994
Ions	meq L^-1^	
Ca^2+^		1.867
Mg^2+^		0.793
Na^+^		5.020
K^+^		1.812
Cl^-^		5.890
HCO3^-^		1.840
SO_4_^2-^		1.762
Cation Exchange Capacity (CEC)	c mol kg^-1^	44.38
Available nutrients		
N	mg Kg^-1^	42.67
P		8.01
K		234.58
Zn		4.36
Cu		7.95
Pb		4.14
Total nutrients		
N	g Kg^-1^	1.020
P		1.289
K		2.712
Zn	mg Kg^-1^	226.8
Cu		66.11
Pb		82.46
C	%	0.827
O.M		1.425
C:N ratio		8.11
Soil Bulk Density (BD)	g cm^-3^	1.63
Particle size distribution		
Clay (less than 0.002 mm)	%	46.98
Silt (2.0 mm – 0.05 mm)		31.22
Sand (0.05 mm – 0.002 mm)		21.80
Texture		Clay

### 2.1. Description of the selected studied soils

**Fig 1:**
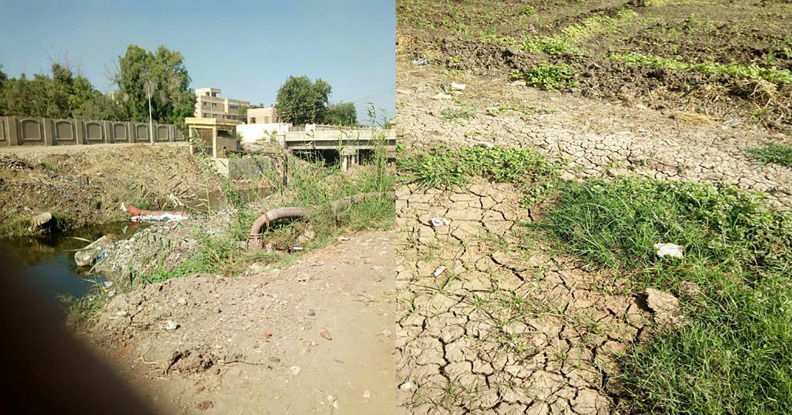
Study location of polluted soil.

Soil samples (0–30 cm depth) used in this experiment were collected and homogenized from the Kafr El-Zayat region of the Gharbia Governorate in Egypt. As shown in Fig 1, irrigation using from the Tala drainage canal is the primary source of pollution in the study area. According to the USDA soil taxonomy, the soils are classified as *Vertic Torriuvents* (Entisols order) [42].

### 2.2. Azolla and compost production

#### 2.2.1. Azolla growing.

**Fig 2:**
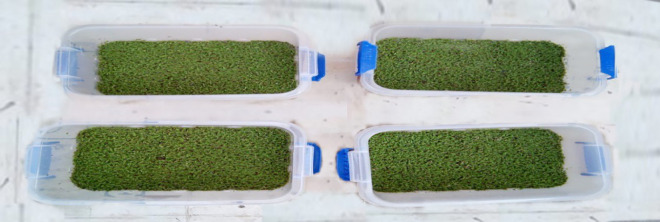
Photographic photo illustrate *Azolla pinnata* which used in the experiment.

The Azolla (*Azolla pinnata*) was obtained from the National Agricultural Research Center in Giza. The plants were cultivated in plastic tanks (20 cm h × 40 × 25) (Fig 2), filled with 12 L of water under controlled temperature and nutritional conditions to ensure optimal quality experimentation. Each tank was inoculated with 100 g of *A. pinnata*. The azolla was harvested after two weeks, once it formed a dense mat covering the entire water surface, and this biomass was then applied to the treatment pots.

#### 2.2..2. Media used and climate data.

Yoshida medium [43] was used to grow *A. pinnata*. This medium contained the following chemical compositions in ppm: 40.00 mg L^-1^ NaH_2_PO_4_.H_2_O, 40.00 mg L^-1^ K_2_SO_4_, 40.00 mg L^-1^ CaCl_2_, 40.00 mg L^-1^ MgSO_4_.7H_2_O, 0.50 mg L^-1^ MnCl_2_. 2H_2_O, 0.15 mg L^-1^ NaMoO_3_.2H_2_O, 0.20 mg L^-1^ H_3_BO_3_, 0.01 mg L^-1^ ZnSO_4_.7H_2_O, 0.01 mg L^-1^ CuSO_4_.5H_2_O, 2.00 mg L^-1^ Iron (Fe-EDTA) and pH was adjusted to 5.5. Mediterranean weather dominates the climate in the study area, with hot, dry summers and moderate winter rainfall [44]. Summer temperatures typically range between 32°C and 38°C, occasionally reaching 45°C in July and August, with relatively high relative humidity [45].

#### 2.2.3. Azolla compost preparation.

Azolla (*A. pinnata*) was collected and prepared according to Jumadi et al., [35]. Five kilograms of *A. pinnata* was collected from the National Agricultural Research Center in Giza and cleaned, washed, and dried in the sun for 2 days until a stable moisture content of approximately 50% was achieved. Then mixed with 250 ml of molasses and placed in a black plastic pack and covered with a second black plastic sheet. Water was added during composting to maintain the moisture at 50–60% of the total weight. The composting process spanned 2 weeks. Table 2 showed some chemical properties of *A. pinnata* and azolla compost.

**Table 2:** Some characteristics of two forms of azolla used in this study.

Properties	Unit	*Azolla pinnata*	Azolla Compost
pH (1:10)		---	7.38
EC (1:10)	dS m^-1^	---	2.78
Total N	%	4.32	4.62
Total P	%	1.26	1.41
Total K	%	1.42	1.63
Total C	%	49.86	38.52
C:N ratio		11.54	8.33

### 2.3. Experimental design

During the summer of 2021, a pot experiment was conducted with soil samples sieved through a 4 mm sieve to reflect the natural soil condition. For the thirteen treatments, ten kilograms of soil were weighed and placed into each of the 65 pots. This total included five pre-treated pots and the control. Compost was applied and incorporated according to the treatment before rice transplantation. The experiment followed a randomized complete block design with five replications. The Complete Block Randomized Design (CBRD) was used to control variability caused by environmental or spatial heterogeneity, which is common in pot experiments. By grouping similar experimental units into blocks and randomly assigning treatments within each block, the influence of uncontrolled variability (e.g., differences in light, temperature, or soil across the layout) was minimized. This design increases the precision of treatment comparisons and ensures that the observed effects are primarily attributable to the treatments rather than to extraneous environmental variation.

On June 12, 2021, ten Sakha 104 variety seedlings were transplanted in each pot; 15 days after sowing, the plants were thinned to five seedlings per pot, and they were harvested 145 days after sowing.

**Fig 3:**
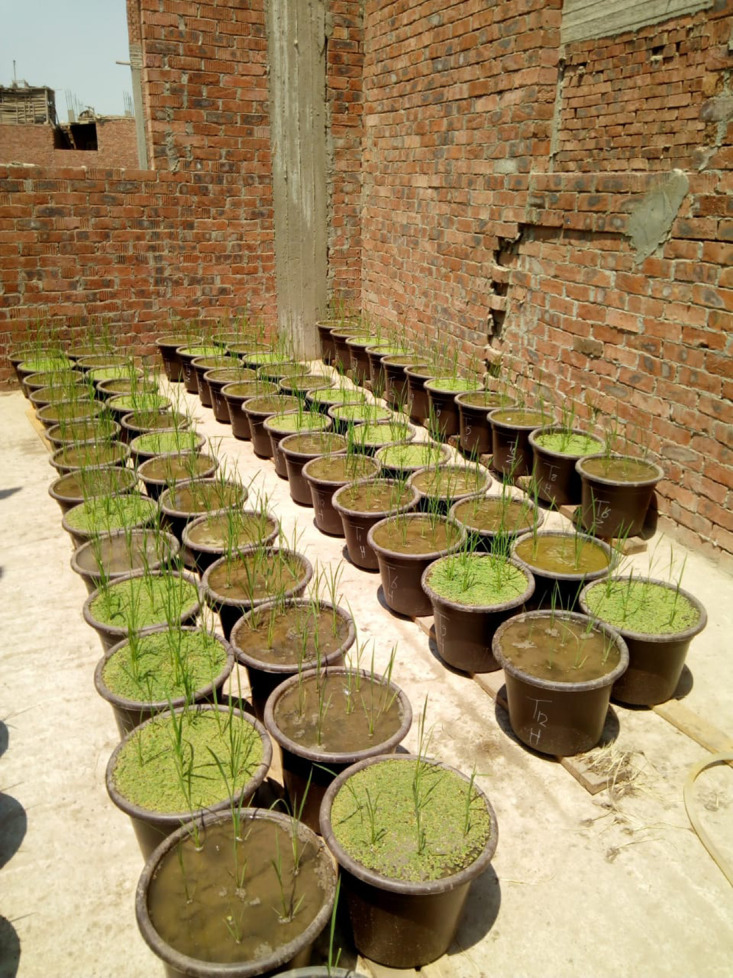
Photographic photo for the experiment.

Rice seeds were soaked in fresh water for 24 hours, rinsed, and then incubated for 48 hours to speed up early germination in order to prepare the rice nursery. After 30 days of growth in the occupation, the seedlings were carefully removed and transplanted into the experimental pots, with ten seedlings placed in each pot. Ten seedlings were handled per pot. *A. pinnata* was inoculated at the designated rate five days after transplanting, whereas azolla compost was incorporated into soil prior to the transplanting as presented in Fig 3. It is recommended that 240 kg ha^-1^ of phosphorus be added before cultivation as superphosphate (15.5% P_2_O_5_) and potassium be added at a rate of 120 kg ha^-1^ as potassium sulphate (48% K_2_O). In regards to the nitrogen treatments, the recommended dose of N fertilizer for Sakha 104 is 480 kg ha^-1^ as ammonium sulphate (21.5%). The other agricultural practices were done as the recommendation of Ministry of Agriculture. A list of thirteen studied treatments in this experiment were as follows: T1: Control (without any soil applications), T2: NPK fertilizer (Recommended dose), T3: 100%NPK + 5 Mg ha^-1^ *A. pinnata*, T4: 100%NPK + 10 Mg ha^-1^ *A. pinnata*, T5: 100%NPK + 7 Mg ha^-1^ azolla compost, T6: 100%NPK + 10 Mg ha^-1^ azolla compost, T7: 100%NPK + 14 Mg ha^-1^ azolla compost,T8:50%N + PK + 5 Mg ha^-1^ *A. pinnata*, T9: 50%N + PK + 10 Mg ha^-1^ *A. pinnata*, T10: 50%N + PK + 7 Mg ha^-1^ azolla compost, T11:50%N + PK + 10 Mg ha^-1^ azolla compost, T12:50%N + PK + 14 Mg ha^-1^ azolla compost, T13:100%NPK + 5 Mg ha^-1^ *A. pinnata* +7 Mg ha^-1^ azolla compost.

At harvest, data were collected on grain yield, straw biomass, and 1000-grain weight. Each pot's plants were cut 5 cm above the soil surface, air-died, and then threshed to separate grains from straw. Grain and straw yields were calculated and expressed as Mg ha ⁻ ¹. Subsequently, plant samples were prepared for analysis based on these results. Following the rice harvesting, soil samples were collected from each pot for laboratory analysis.

### 2.4. Experimental analytical procedures

After 145 days, plants were harvested from each pot, and rice grain and straw yields were determined. The plant samples were transported to the laboratory, cleaned with distilled water to remove surface dust and debris and air-dried. The samples were sealed in paper bags oven- dried at 70 °C for 24 hours.

Samples were ground in the sample prior to analysis. The wet digesting method was used to digest plant materials using a solution of hydrogen peroxide and concentrated sulfuric acid [46]. Phosphorus, potassium, and total nitrogen were analyzed in the digests. The Kjeldahl method, as explained by Page et al., [47], was used to determine total nitrogen (N). Using a spectrophotometer (wavelength 880 nm, UV-VIS 752N j) the amount of total phosphorus was determined [48]. According to Jackson [49], a flame photometer (model PFP7) was used to measure potassium (K+). While pH was evaluated in a 1:2.5 suspension, the electrical conductivity (Orion, Model 150, USA) of the soil was determined in a 1:5 extract. A pH meter (from HANNA Instruments (Leighton Buzzard, UK) and conductivity meter (Orion,Model150,USA) were used to assess the EC and pH of azolla compost in a 1:10 (w:v) water-soluble extracts [50].

Cations (Na^+^, K^+^, Ca^2+^, and Mg^2+^) and anions (CO_3_^2-^, HCO_3_^-^ and Cl^-^) were determined in a 1:5 extract as described by Page et al., [47]. Sulfate anions (SO_4_^2-^) were calculated as the difference between soluble cations and anions. Soil calcium carbonate content was determined by using a calcimeter [51]. The method of Na^+^ saturation using 1M sodium acetate solution (pH 8.2), followed by ethanol rinsing and substituting adsorbed Na^+^ by NH_4_^+^ using 1M ammonium acetate solution (pH 7.0), was used to estimate CEC [52]. Following extraction by 0.5 M NaHCO_3_ solution at pH 8.30, available P was measured using a spectrophotometer utilizing the ascorbic acid method [53]. Available N was evaluated using the Kjeldahl method after extraction by 2M KCl solution [54]. Available potassium of soil was extracted with 1N ammonium acetate and determined using a flame photometer as described by Cottenie et al., [46]. The total N, P, and K were measured according to Sparks [55]. Soil bulk density of the undisturbed soil sample was measured using the core method as explained by Grossman et al., [56]. Particle size distribution was determined using the international pipette method, as described by Tan et al., [57]. Organic matter of azolla compost was calculated as the difference between ash and dry weight (ash content was determined in a muffle oven at 550°C for 8h); 50% of organic matter was considered organic C [58]. Soil organic carbon was measured using the Walkley-Black method with the wet digestion process by 1 N potassium dichromate (K_2_Cr_2_O_7_) solution and concentrated sulfuric (H_2_SO_4_) acid [59], from where organic matter was calculated.


Organic matter (%) = organic carbon (%) ×1.724 (1)

### 2.5. Statistical analysis

The experiment was arranged in a randomized complete block design with five replications. All data presented was prepared for statistical analysis. SAS software was used to statistically evaluate all the data that was supplied. The means of the treatments were compared using Duncan's multiple range tests. A P < 0.05 level of statistical significance was applied.

## 3. Results and discussion

### 3.1. Effects of different treatments on rice yield and yield components

The grain yield, straw yield, and 1000-grain weight of rice for the various treatments are presented in Table 3. Generally, all treatments resulted in significantly higher yields than the control. The greatest increase was observed for treatment T13, which combined *A. pinnata* as a green manure and its compost, producing significantly higher grain and straw yields than any individual amendment. Grain and straw yields increased steadily with the higher application rates of both *A. pinnata* and azolla compost.

**Table 3:** Effects of different treatments on grain, straw yield of rice and 1000-grain weight and their relative change.

Treatments	Grain Mg ha^-1^		Straw Mg ha^-1^		1000-grain weight g	
	Relative Change (%)	Mean ±Std Error	Relative Change (%)	Mean ±Std Error	Relative Change (%)	Mean ±Std Error
T1	6.26^H^ ±0.25	----	6.96^G^ ±0.13	----	18.40^I^ ±0.14	----
T2	8.55^E^ ±0.07	**+**36.58	10.56^D^ ± 0.06	**+**51.72	21.48^EF^ ±0.15	**+**16.74
T3	9.72^B^ ±0.12	**+**55.27	11.10^C^ ± 0.09	**+**59.48	23.72^BC^ ± 0.02	**+**28.91
T4	10.08^AB^ ± 0.11	**+**61.02	12.44^B^ ± 0.20	**+**78.74	24.72^AB^ ± 0.09	**+**34.35
T5	8.74^DE^ ±0.10	**+**39.62	10.38^D^ ± 0.26	**+**49.14	22.39^DE^ ± 0.30	**+**21.68
T6	9.09 CD ±0.11	**+**45.21	10.65^D^ ± 0.17	**+**53.02	23.19 CD ± 0.02	**+**26.03
T7	9.19^C^ ±0.25	**+**46.81	11.16^C^ ± 0.11	**+**60.34	23.54^C^ ±1.17	**+**27.93
T8	8.45^E^ ±0.16	**+**34.98	10.40^D^ ± 0.17	**+**49.43	21.16^FG^ ± 0.03	**+**15.00
T9	8.59^E^ ±0.12	**+**37.22	10.56^D^ ± 0.17	**+**51.72	21.66^EF^ ± 0.08	**+**17.72
T10	7.46^G^ ±0.12	**+**19.17	9.24^F^ ±0.10	**+**32.76	19.93^H^ ±0.05	**+**8.32
T11	7.92^F^ ±0.18	**+**26.52	9.86^E^ ±0.13	**+**41.67	20.42^GH^ ± 0.08	**+**10.98
T12	8.45^E^ ±0.12	**+**34.98	10.38^D^ ± 0.16	**+**49.14	20.77^FGH^ ± 0.09	**+**12.88
T13	10.26^A^ ±0.17	**+**63.90	12.92^A^ ± 0.12	**+**85.63	25.52^A^ ±0.41	**+**38.70
L.S.D _0.05_	0.4433	--	0.4328	--	1.0329	--

T1 → Control (without any applications), T2 → NPK fertilizer (Recommended), T3 → NPK + 5 Mg ha^-1^ *A. pinnata* azolla, T4 → NPK + 10 Mg ha^-1^ *A. pinnata*, T5 → NPK + 7 Mg ha^-1^ azolla compost, T6 → NPK + 10 Mg ha^-1^ azolla compost, T7 → NPK + 14 Mg ha^-1^ azolla compost,T8 → 50%N + PK + 5 Mg ha^-1^ *A. pinnata*, T9 → 50%N + PK + 10 Mg ha^-1^ *A. pinnata*, T10 → 50%N + PK + 7 Mg ha^-1^ azolla compost, T11 → 50%N + PK + 10 Mg ha^-1^ azolla compost, T12 → 50%N + PK + 14 Mg ha^-1^ azolla compost, T13 → NPK + 5 Mg ha^-1^ *A. pinnata* +7 Mg ha^-1^ azolla compost.

Means within a Colum followed by the same letter are not statistically different by LSD at 0.05 level.

Regarding the form of amendment, azolla, *A. pinnata*, produced higher grain and straw yields than corresponding treatments of azolla compost. High grains yield(10.26 Mg ha^-1^) was obtained from the treatment combining *A. pinnata* and its compost under the recommended NPK fertilizer dose, significantly outperforming all other treatments. The application of *A. pinnata* to the soil at 5 and 10 Mg ha^-1^ increased grain yield by 55.27% and 61.02%, respectively. While the corresponding increases for azolla compost applied at 7, 10 and 14 Mg ha^-1^ were 39.62%, 45.21%, 46.81%, respectively. There is limited information regarding the application of azolla compost as a soil amendment in rice production systems. A previous study reported maximum rice grain yields of rice with combined application of 80 N kg ha^-1^ and 5 Mg ha^-1^of azolla compost [60]. Our results are in agree with those of [61], which reported that azolla application increased rice growth and yield by 15.54%, 25.49%, respectively. The same trend was observed for straw yield; all treatments significantly increased straw yield. The highest straw yield (85.63% greater than the control) was recorded in treatment T13,which amended the soil with 5 Mg ha^-1^ of *A. pinnata* azolla and 7 Mg ha^-1^ of azolla compost along with the full recommended rate of NPK fertilizer. The 1000-grain weight was highest in treatment T13(38.7% greater than the control), which received 5 Mg ha^-1^ of *A. pinnata* and 7 Mg ha^-1^ of azolla compost along with the full recommended rate of NPK fertilizer.

The application of azolla as *A. pinnata* or compost significantly increased grain and straw yield. The improvement in growth was likely due to enhanced soil conditions and a reduction in toxic heavy metal availability. In this experiment, the combined treatment of 100% NPK + 5 Mg ha^-1^ *A. pinnata* and 7 Mg ha^-1^ azolla compost(T13) was the most effective. As a result, the integration of NPK fertilizer with *A. pinnata* and azolla compost forms successfully increased rice productivity. Treatments combining NPK with only one form of azolla were less effective. The findings in Table 3 indicate that *A. pinnata* had more noticeable effect than azolla compost on rice yield.. The combination of NPK fertilizer with *A. pinnata* produced a grater response than its combination with azolla compost. This enhanced performance with integrated *A. pinnata* and synthetic fertilizer application may be attributed to improved nitrogen use efficiency, resulting from reduced nitrogen loss and increased nitrogen uptake by the rice plants [62].

Globally, the most crucial objectives in rice production are increase grain yield and farmer income while reducing production costs and minimizing environmental pollution risks [63]. Therefore, it is vital to use existing mechanisms, such as phytoremediation. When grown in a rice field azolla, provides nitrogen to the crop, reduces water temperature and soil pH, inhibits NH_3_ volatilization, and preventing weeds and mosquitoes growth [64]. The data demonstrate that both either *A. pinnata* and azolla compost, can increase rice yield. these results are consistent with previous studies on biofertilizer applications in rice [65], Faba beans [66],and maize [67],which reported similar yield enhancements.

### 3.2. Effects on NPK uptake by rice grains and straw

At the 0.05 level of probability, the increase in NPK uptake was statistically significant. uptake was higher in treatments of *A. pinnata* and its compost under full NPK recommendation than in either amendment alone. Furthermore, N, P, and K uptake in grains increased with higher application rates of *A. pinnata* compared to azolla compost (Table 5). As shown in Table 5, nitrogen uptake by rice improved with the application of NPK fertilizer and/or azolla amendments. Uptake increased progressively with higher application rates of these treatments. Compared to the control, *A. pinnata* application increased grain N uptake by 243.7%; this increase was 358.1% relative to azolla compost applications. The combined treatment of a high azolla compost rate with the full NPK recommendation enhanced N uptake by 244.6%.

When compared to other treatments in soils, the NPK + 5 Mg ha^-1^ *A. pinnata* +7 Mg ha^-1^ azolla compost treatment resulted in a 260.1% increase in P uptake by grains. When *A. pinnata* was applied at rates of 5 and 10 Mg ha^-1^, P uptake rose by 184.6% and 230.9%, respectively. P absorption rose by 162.5% when azolla compost was applied at a high rate and NPK fertilizer was fully recommended.

The data in Table 4 showed that the K uptake in rice grains grown in contaminated soil increased significantly. Treatment 13 (T13) (NPK + 5 Mg ha^-1^ *A. pinnata* +7 Mg ha^-1^ azolla compost) resulted in the highest K uptake in grains, at approximately 264.2%. *A. pinnata* treatment at rates of 5 and 10 mg ha^-1^, on the other hand, enhanced K uptake by 178.3% and 223.2%, respectively. K uptake rose by 164.8% when azolla compost was applied at a high rate and NPK fertilizer was fully recommended. *A. pinnata* and its compost treatments with full NPK fertilizer recommendations (T13) had higher NPK uptake than either *A. pinnata* or azolla compost treatments alone. This increase in NPK uptake was statistically significant at the 0.05 level of probability (Table 4). While the application of azolla compost at a high rate with full NPK fertilizer prescription enhanced N uptake by 215.4%, Table 5 shows that the application of *A. pinnata* at rates of 5 and 10 Mg ha^-1^ boosted straw N uptake by 200.8% and 267.3%, respectively. The 100% NPK + 5 Mg ha^-1^ *A. pinnata* +7 Mg ha^-1^ azolla compost treatment resulted in a 333.3% higher P absorption in soils than other treatments. The application of 5 and 10 Mg ha ⁻ ¹ of *A. pinnata* increased P uptake by 195.2% and 280.8%, respectively. P absorption increased by 206.3% when azolla compost was applied at a high rate and NPK fertilizer was fully recommended (Table 4). Treatment 13 (NPK + 5 Mg ha^-1^ *A. pinnata* +7 Mg ha^-1^ azolla compost resulted in the highest K uptake in grains, at approximately 268.9%. In contrast, *A. pinnata* applied at rates of 5 and 10 Mg ha^-1^ boosted K uptake by 141.1% and 226.5%, respectively. When azolla compost was applied at a high rate and NPK fertilizer was fully recommended, K absorption increased by 147.6% (Table 4).

**Table 4:** Effects of different treatments on NPK uptake by rice grains and straw and their relative change.

Treatments	NPK uptake by rice straw						NPK uptake by rice grains					
	K uptake Kg ha^-1^		P uptake Kg ha^-1^		N uptake Kg ha^-1^		K uptake Kg ha^-1^		P uptake Kg ha^-1^		N uptake Kg ha^-1^	
	Relative Change (%)	Mean ±Std Error	Relative Change (%)	Mean ±Std Error	Relative Change (%)	Mean ±Std Error	Relative Change (%)	Mean ±Std Error	Relative Change (%)	Mean ±Std Error	Relative Change (%)	Mean ±Std Error
T1	34.52^H^	----	12.48^J^	----	13.33^J^	----	22.48^I^	----	10.85^J^	----	67.59^I^	----
	±1.544		± 0.42		±0.73		±0.64		±0.55		±2.72	
T2	72.28^F^	**109.3**	27.62^F^ ± 0.50	**121.3**	29.20^F^	**119**	54.27^EF^±1.30	**141.4**	28.96^EF^ 0.87	**166.9**	127.85^EF^±0.42	**89.15**
	±0.79				±0.45							
T3	118.67^C^	**243.7**	35.53^C^	**184.6**	37.10^C^	**178.3**	67.62^C^	**200.8**	32.04^CD^±0.68	**195.2**	163.0^C^	**141.1**
	±5.88		±0.37		±0.44		±1.92				±2.65	
T4	158.14^B^	**358.1**	41.30^B^	**230.9**	43.09^B^	**223.2**	82.59^B^	**267.3**	41.32^B^±0.53	**280.8**	220.7^B^	**226.5**
	±2.16		±0.36		±0.82		±2.91				±3.73	
T5	92.78^E^	**168.7**	30.01^E^	**140.4**	31.24^E^	**134.3**	57.93^DE^±1.65	**157.6**	28.82^EF^±0.48	**165.6**	117.1^GH^±2.79	**73.25**
	±2.94		±061		±0.41							
T6	105.04^D^	**204.2**	31.90^DE^	**155.6**	33.58^D^	**151.9**	59.23^D^	**163.4**	30.22^DE^±0.79	**178.5**	139.8^D^	**106.8**
	±1.34		±0.64		±0.76		±0.68				±2.56	
T7	118.99^C^	**244.6**	32.77^D^	**162.5**	35.30^CD^	**164.8**	70.91^C^	**215.4**	33.24^C^	**206.3**	167.4^C^	**147.6**
	±7.67		±1.57		±1.22		±1.11		±0.64		± 1.35	
T8	69.11^F^	**100.2**	25.45^GH^	**103.9**	26.62^GH^	**99.69**	52.12^FG^±0.95	**131.8**	25.73^G H^ ± 0.79	**137.1**	122.6^FG^±3.12	**81.38**
	± 0.90		±1.05		±0.60							
T9	72.43^F^	**109.8**	26.54^FG^	**112.6**	28.55^FG^	**114.1**	53.34^F^	**137.2**	27.42^FG^±0.37	**152.7**	131.88^E^±3.06	**95.11**
	±1.55		±0.48		±0. 90		±1.67					
T10	60.24^G^	**74.5**	21.78^I^	**74.51**	23.38^I^	**75.39**	44.89^H^ ± 0.18	**99.68**	22.89^I^	**110.9**	110.2^H^	**63.04**
	±1.00		±0.24		± 0.49				±0.40		±1.20	
T11	72.54^F^	**110.1**	24.15^H^	**93.5**	25.08 ^HI^	**88.14**	49.13^G^	**118.5**	24.85^H^	**129**	119.8^G^	**77.24**
	±1.71		±0.71		±0.78		±0.90		±0.81		±2.65	
T12	108.94^D^	**215.5**	25.95^FGH^	**107.9**	27.42^FG^	**105.7**	53.59^F^	**138.3**	26.53^G H^	**144.5**	133.5^DE^±1.88	**97.51**
	±0.63		±0.24		±0.63		±1.41		± 0.36			
T13	174.68^A^	**406**	44.95^A^	**260.1**	48.55^A^	**264.2**	91.95^A^	**309**	47.02^A^	**333.3**	249.4^A^	**268.9**
	±2.50		±0.51		±0.52		±0.82		±0.71		±4.60	
L.S.D _0.05_	8.8803	--	1.9707	--	2.0305	--	4.0369	--	1.827	--	7.8215	--

**Table 5:** Effects of different treatments on Zn, Cu and Pb rice uptake and concentration in grains and their relative change.

Treatments	Zn, Cu and Pb concentration						Zn, Cu and Pb uptake					
	Pb g ha^-1^		Cu g ha^-1^		Zn g ha^-1^		Pb g ha^-1^		Cu g ha^-1^		Zn g ha^-1^	
	Relative Change (%)	Mean ±Std Error	Relative Change (%)	Mean ±Std Error	Relative Change (%)	Mean ±Std Error	Relative Change (%)	Mean ±Std Error	Relative Change (%)	Mean ±Std Error	Relative Change (%)	Mean ±Std Error
T1	278.611^I^	----	101.82^H^	----	79.417^F^	----	44.490^A^	----	16.272^A^	----	12.662^A^	----
	±10.97		±3.46		±4.21		±0.07		±0.17		±0.16	
T2	379.56^DEF^	36.21	137.04^BCD^	34.59	107.015^BC^	34.75	44.387^A^	-0.23	16.026^AB^	-1.51	12.515^A^	-1.16
	**±**3.18		±1.69		±1.59		±0.06		±0.16		±0.17	
T3	415.40^BC^	49.09	142.76^ABC^	40.2	118.108^A^	48.71	42.722^EF^	-3.97	14.678^GH^	-9.79	12.151^AB^	-4.03
	±5.68		±3.17		±1.88		±0.10		±0.15		±0.22	
T4	424.98^AB^	52.53	144.957^A^	42.36	116.942^A^	47.25	42.140^G^	-5.28	14.370^HI^	-11.68	11.598^BC^	-8.4
	±4.94		±2.78		±3.25		±0.14		±0.15		±0.33	
T5	376.67^EF^	35.19	132.42^DE^	30.05	107.078^BC^	34.83	43.097^D^	-3.13	15.155^EF^	-6.86	12.245^AB^	-3.29
	±4.26		±0.92		±3.21		±0.10		±0.16		±0.25	
T6	398.141^CD^	42.9	136.12^CDE^	33.68	110.62^ABC^	39.29	43.780^B^	-1.59	14.970^FG^	-8	12.169^AB^	-3.89
	±5.19		±1.59		±2.05		±0.25		±0.15		±0.26	
T7	395.60^DE^	41.99	130.661^DE^	28.32	105.34^BCD^	32.64	42.995^DE^	-0.42	14.193^I^	-12.77	11.428^C^	-9.74
	±11.29		±4.59		±5.25		±0.07		±0.15		±0.28	
T8	374.638^F^	34.46	134.100^DE^	31.7	105.62^BCD^	32.99	44.300^A^	-0.51	15.863^ABC^	-2.51	12.486^A^	-1.38
	±6.72		±1.54		±2.69		±0.08		±0.16		±0.14	
T9	380.5^DEF^	36.57	135.203^DE^	32.78	106.58^BCD^	34.2	44.262^A^	-1.4	15.729^BCD^	-3.33	12.386^A^	-2.17
	±4.85		±1.17		±3.47		±0.07		±0.16		±0.23	
T10	327.513^H^	17.55	116.386^G^	14.3	92.100^E^	15.97	43.867^B^	-1.47	15.59^BCDE^	-4.19	12.333^A^	-2.59
	±4.92		±1.51		±1.85		±0.06		±0.16		±0.09	
T11	347.483^G^	24.72	122.49^FG^	20.3	97.584^DE^	22.87	43.835^B^	-2.34	15.451^CDE^	-5.04	12.321^A^	-2.69
	±8.48		±3.39		±1.41		±0.13		±0.16		±0.19	
T12	367.422^F^	31.87	129.44^EF^	27.12	103.62^CD^	30.48	43.447^C^	-31.87	15.304^DEF^	-5.94	12.257^AB^	-3.19
	±4.82		±2.41		±2.09		±0.12		±0.16		±0.26	
T13	435.789^A^	56.41	143.842^A^	41.27	114.149^AB^	43.73	42.467^F^	-56.41	14.020^I^	-13.83	11.117^C^	-12.2
	±7.10		±2.11		±4.92		±0.10		±0.15		±.40	
L.S.D _0.05_	19.412	--	7.3144	--	9.0394	--	0.3226	--	0.4491	--	0.6963	--

Consistent with conclusion, it found that the greatest N, P and K uptake occurred when azolla at two forms and NPK were applied together. This behavior has been observed and discussed a great deal in literature. For instance, azolla can thrive in flooded rice fields [68]. It is also widely utilized as a highly effective biofertilizer for rice fields, increasing their nitrogen content within a few weeks of application. Azolla contains a number of vitamins, including beta-carotene, vitamin A, and vitamin B-12, and is a good source of protein and essential amino acids (nitrogen 4%–5%, phosphorus 0.5%–0.9%). Both macro- and micronutrients, including calcium, phosphorus, potassium, magnesium, copper, zinc, and others, are abundant in it. Furthermore, the protein content of azolla ranges from 25% to 35% on a dry weight basis. The reason for the increase in N uptake may be the role of azolla application in increasing the nitrogen content of the soil solution, enhancing its absorption by the roots and facilitating its transfer to the vegetative parts of the plant [69]. The increase in the nitrogen and phosphorous content of rice components as a result of appropriate levels phosphate and nitrogen fertilizers may be attributed to the role of these fertilizers in improving the physical, chemical and biological characteristics of the soil. This improvement may have been positively reflected in an increase in the soil solution’s content of plant nutrients, thereby increasing their availability in the root zone (rhizosphere). Additionally, it may have enhanced the growth of the root system and its propagation capacity, which led to an increase roots absorption capacity and raised the efficiency uptake and their transfer to different plant tissues including leaves, consequently increasing their content of these elements [70].

Composting Azolla leads to mineralization of organic matter, resulting in higher content of available nutrients. The effects of azolla compost treatments on nutrient uptake in rice grain and straw sections have been extensively studied in the past. According to one of these studies, Zadeh [71], plots treated with azolla compost at a rate of 5 t ha^-1^ accumulated the most nitrogen (N) in both the grain and straw. Additionally, adding azolla compost (at a rate of 5 t ha^-1^) increased the amount of available nitrogen and phosphorus (N and P) in the farmed soil, which was positively reflected in the increased content of both nutrients inside paddy plants [72]. Similar to this study's findings, other researchers [73] found that the combined use of synthetic and biological fertilizers led to a progressive increase in N uptake (up to 200 kg ha^-1^). Additionally, Das et al. [74] reported that applying azolla compost together with half the rate of synthetic fertilizer (5 t ha^-1^) produced better soil fertility and a notable increase in NPK uptake values in maize plants compared to applying recommended chemical fertilizer doses.

Compared to applying urea alone, adding azolla significantly increased rice plant N absorption and N-use efficacy while lowering N loss [75]. However, compared to urea-only release, azolla compost can provide a continuous and gradual release of soil ammoniacal nitrogen (NH^+^-N) and nitrate-nitrogen (NO3-N) [35,76]. In general, adding organic fertilisers leads to better NPK nutrient uptake; on the other hand, applying synthetic fertilizers (in the entire recommended dosage) is a consequence of attaining NPK status in plants and their cultivated soil. Additionally, using organic fertilizers can significantly reduce the amount of synthetic fertilizer needed [77–79].

According to estimates, the *A. azollae* system has a nitrogen fixation capability of 1.1 kg N ha^-1^ day-1. In roughly 20–25 days, one azolla crop supplied 20–40 kg N ha^-1^ to the rice crop [80].

Similar findings and conclusions have been published by numerous others. For example, Raja [81], reported that azolla biomass can be utilized in rice fields to partially or fully replace synthetic fertilizers, as it can supply 1.5–2.0 million tons of nitrogen, while 3.3–4.0 million tons of urea are needed for the same amount of crop yield. According to Setiawati et al., [80] applying *A. pinnata* in both fresh and powdered form increases the amount of nitrogen in rice production by up to 3–5%. According to Raja et al., [81], azolla treatment of rice plants may improve nitrogen uptake and availability [82]. The plants’ capacity to absorb nitrogen was enhanced by the availability of nitrogen in proportion to their requirements and developmental phases. The improvement of rice growth, internode elongation, photosynthesis, metabolism, and assimilation production may be the cause of this [83]. The increase in the plants’ ability to directly absorb and use nitrogen had an impact on plant growth and productivity, as plants that were able to develop optimally during the vegetative phase were able to grow even more during the generative phase. According to the study's findings, azolla compost may be able to assist in restoring the soil's proper nutritional levels when synthetic fertilizer additions are insufficient, as in the T10, T11, and T12 treatments. The advanced role of azolla compost under lower fertilizer levels may be due to its abundance of various nutrients (such as N and P nutrients), which provide organic matter, exchangeable cations, and gradual delivery [38,84,85].

The maximum accumulation levels of N absorption in both grain and straw components were found in plots treated with azolla compost at a rate of 5 t ha^-1^ [71]. Additionally, according to Setiawati [80], adding azolla compost to cultivated soil at a rate of 5 t ha^-1^ increased the amount of available nitrogen and phosphorus (N and P), which was reflected favorably in the increased nutrient content of paddy plants.

### 3.3. Effects on Zn, Cu and Pb uptake by rice grains

As seen in Table 5, the absorption of zinc (Zn), copper (Cu), and lead (Pb) in rice grains increased dramatically with each treatment when compared to the control. However, the concentration of these components in grains declined. The increase in the uptake of these elements was due to the diluting effect caused by the increase in rice yield. *A. pinnata* treated with 5 and 10 Mg ha^-1^ enhanced zinc uptake by 49.09% and 52.53%, respectively. Zn uptake increased by 41.99% when azolla compost was applied at a high rate and NPK fertilizer was fully recommended. When compared to other treatments in soils, the combination of NPK + 5 Mg ha^-1^ *A. pinnata* and 7 Mg ha^-1^ azolla compost resulted in a 41.27% increase in Cu uptake by grains. Using *A. pinnata* at rates of 5 and 10 Mg ha^-1^ resulted in a 40.20% and 42.36% increase in Cu absorption, respectively. Cu uptake increased by 28.32% when azolla compost was applied at a high rate and NPK fertilizer was fully recommended (Table 5). Treatment 3 (azolla application at rate 5 Mg ha^-1^) resulted in the highest Pb uptake in grains, at approximately 48.71%. When azolla compost was applied at a high rate and NPK fertilizer was fully recommended, Pb uptake increased by 32.64%.

### 3.4. Effects on Zn, Cu and Pb concentration in rice grains

As indicated in Table 5, the concentrations of zinc (Zn), copper (Cu), and lead (Pb) in rice grains dropped dramatically with each application when compared to the control treatment, with the exception of zinc in treatments that applied azolla compost and the recommended NPK fertilizer under contamination conditions. *A. pinnata* applications of 5 and 10 Mg ha^-1^ reduced the content of zinc by 3.97% and 5.28%, respectively. Applying azolla compost at a high rate while fully recommending NPK fertilizer in contaminated soils reduced the concentration of zinc by 0.42%. However, the control treatment resulted in a significant concentration of copper in the grains. In contaminated soil, Treatment 13 had the lowest concentration value (13.83%) when compared to the control treatment. The content of Cu was reduced by 9.79% and 11.68%, respectively, by applying *A. pinnata* at rates of 5 and 10 mg ha^-1^. Cu concentration was reduced by 12.77% when azolla compost was applied at a high rate and NPK fertilizer was fully recommended. When azolla compost was applied at a high rate and NPK fertilizer was fully recommended, the Pb concentration dropped by 9.74%. It is well known that the addition of organic matter leads to a reduction soil pH, which may increase the availability of heavy metals in soil solution and allow azolla roots to absorb these elements from soil. These conditions help reduce the concentration of heavy metals in the soil solution and in growing plants such as rice. on the other hand, the absorption of these element by azolla will increase, which was the objective of our study.

Since rice is a hyper-accumulator plant that can store heavy metals without experiencing physiological disturbance, the results of the effect of azolla application on Zn, Cu, and Pb concentration are in good agreement with those previously reported by numerous authors [31,86]. This is particularly dangerous because rice is a staple food. In fact, eating rice was thought to be the main way that people were exposed to heavy metals [87]. The study found that Pb content in grain decreased significantly with the application of *Azolla microphylla* with husk biochar (A2B1) at a rate of 9.13 ppm, but not significantly with *A. microphylla* without biochar (A2B0) at a rate of 10.59, which is in line with [88]. The highest Pb concentration in grains was recorded in control treatment, whereas T13 recorded the highest decrement by about 12.20% under contamination condition (Table 5).

### 3.5. Effects of different treatments on some soil chemical properties after rice harvesting

#### 3.5.1. Effect on soil pH.

According to Table 6, azolla prevented the pH from rising. The original pH of the uncontaminated soil was 7.9, while the pH of the contaminated soils was 8.14, as indicated in Table 1. The pH of the contaminated soils decreased considerably with azolla compost and *A. pinnata* application at varying amounts, while T2 increased somewhat. The highest rate of azolla compost treatment, at 14 Mg ha^-1^, was responsible for the biggest reduction of 2.09%. This decrease might be the result of the organic acids (humic acid, glycine, cystein, and amino acid) that were produced during the breakdown of azolla compost playing the same role. Our findings concur with those of [89], who revealed that the decomposition of organic waste and the exudates of microorganisms, such as bacteria and fungi, may be the cause of a lower pH by generating significant amounts of organic acids. The pH drop in soil may be caused by acidic functional groups that are produced when organic manure oxidizes [90]. A drop in soil pH following the application of organic materials was also noted by [91–93]

**Table 6:** Effects of different treatments on soil salinity, pH and soluble cation and anion contents after rice harvesting and their relative change.

Treatments	pH		EC dS m^-1^		Soluble cation and anion contents in soil extract (1:2.5 and 1: 5), (meq L^-1^)													
					SO_4_^2-^		HCO_3_^-^		Cl^-^		K^+^		Na^+^		Mg^2+^		Ca^2+^	
	Relative Change (%)	Mean ±Std Error	Relative Change (%)	Mean ±Std Error	Relative Change (%)	Mean ±Std Error	Relative Change (%)	Mean ±Std Error	Relative Change (%)	Mean ±Std Error	Relative Change (%)	Mean ±Std Error	Relative Change (%)	Mean ±Std Error	Relative Change (%)	Mean ±Std Error	Relative Change (%)	Mean ±Std Error
T1	1.005^F^	---	8.13^AB^	---	1.70^C^	---	0.775^D^	---	4.91^A^	---	1.80^D^	---	6.00^A^	0	1.95^AB^	---	1.24^B^	---
	± 0.03		± 0.10		± 0.26		± 0.05		± 0.12		± 0.16		± 0.22		± 0.15		± 0.41	
T2	1.22^BCDEF^	21.39	8.26^A^	1.59	2.92^A^	71.76	1.00^BCD^	29.03	4.53^AB^	-7.73	1.92^CD^	**6.66**	5.90^AB^	-1.66	2.10^A^	**7.69**	2.38^AB^	91.93
	± 0.05		± 0.08		± 0.11		± 0.08		± 0.05		± 0.28		± 0.21		± 0.06		± 0.51	
T3	1.11^CDEF^	10.44	8.11^ABC^	-0.24	2.7^AB^	58.82	1.75^A^	125.8	4.09 ^AB^	-16.7	2.05^BC^	**13.88**	4.90^BCCD^	18.33	2.00^A^	**2.56**	3.69^A^	197.58
	± 0.04		± 0.01		± 0.73		± 0.25		± 0.39		± 0.21		± 0.37		± 0.14		± 0.55	
T4	1.08^DEF^	7.46	8.13^AB^	0	1.50^C^	-11.76	0.82^CD^	5.8	3.93 ^AB^	-19.95	2.72 ^AB^	**51.11**	4.65^CD^	-22.5	1.80 ^ABC^	**-7.69**	2.53^AB^	104.03
	±0.07		± 0.01		± 0.12		± 0.11		± 0.45		± 0.13		± 0.25		± 0.08		± 0.53	
T5	1.459^AB^	45.17	7.97^C^	-1.96	1.95^ABC^	14.7	0.87^BCD^	12.25	4.35 ^AB^	-11.4	2.22^BCD^	**23.33**	5.40^ABC^	-9.99	1.80^ABC^	**-7.69**	2.20^AB^	77.41
	±0.14		± 0.05		± 0.29		± 0.14		± 0.12		± 0.10		± 0.52		± 0.14		± 0.61	
T6	1.478^AB^	47.06	7.97^C^	-1.96	2.17^ABC^	27.64	1.02^BCD^	31.61	4.04 ^AB^	-17.71	2.15^BCD^	**19.44**	4.70^CD^	-21.66	2.05^A^	**5.12**	2.64^AB^	112.9
	± 0.21		± 0.02		± 0.65		± 0.09		± 0.37		± 0.31		± 0.70		± 0.17		± 1.02	
T7	1.497^A^	48.95	7.96^C^	-2.09	1.80^BC^	5.88	1.15^BC^	48.38	3.88 ^AB^	-20.97	2.45^ABC^	**36.11**	4.65^CD^	-22.5	1.55^**C**^	**-20.51**	3.08^AB^	148.38
	± 0.11		± 0.07		± 0.21		± 0.09		± 0.43		± 0.13		± 0.13		± 0.10		± 0.49	
T8	1.18^CDEF^	17.41	8.08^BC^	-0.61	2.02^ABC^	18.82	1.07^BCD^	38.06	4.06 ^AB^	-17.31	2.15^BCD^	19.44	4.95^ABCD^	-17.5	2.00^A^	**2.56**	2.37 ^AB^	91.12
	± 0.04		± 0.09		± 0.19		± 0.15		± 0.17		± 0.10		± 0.46		± 0.22		± 0.46	
T9	1.22^CDEF^	21.39	8.11^ABC^	-0.24	1.72^BC^	1.17	0.90^BCD^	16.12	4.408 ^AB^	-10.22	2.30^BCD^	**27.77**	4.75^CD^	-20.83	1.75^ABC^	**-10.25**	2.83 ^AB^	128.22
	± 0.07		± 0.04		± 0.19		± 0.07		± 0.09		± 0.25		± 0.39		± 0.15		± 0.46	
T10	1.29^ABCDE^	28.35	8.04^BC^	-1.1	2.85^A^	67.64	0.90^BCD^	16.12	4.33 ^AB^	-11.81	2.12^BCD^	**17.77**	5.90^AB^	-1.66	1.90 ^ABC^	**-2.56**	2.40^AB^	93.54
	± 0.09		± 0.02		± 0.09		± 0.06		± 0.30		± 0.25		± 0.44		± 0.06		± 0.60	
T11	1.31^ABCD^	30.34	8.00^BC^	-1.59	1.72^BC^	1.17	0.80^D^	3.22	4.20 ^AB^	-14.46	2.22^BCD^	**23.33**	5.75^ABC^	-4.16	2.00^A^	**2.56**	1.20^B^	-3.22
	±0.09		± 0.03		± 0.08		± 0.15		± 0.50		± 0.19		± 0.43		± 0.14		± 1.05	
T12	1.37^ABC^	36.31	7.99^BC^	-1.72	2.70^AB^	58.82	0.82^CD^	5.8	4.51 ^AB^	-8.14	2.40 ^BCD^	33.33	4.85^BCD^	-19.16	1.60 ^BC^	**-17.94**	3.98^A^	220.96
	±0.07		± 0.05		± 0.47		± 0.10		± 0.42		± 0.25		± 0.20		± 0.18		± 0.53	
T13	1.043 ^F^	3.78	8.06^BC^	-0.86	1.50^C^	-11.76	1.20^A^	54.83	3.75 ^B^	-23.62	3.05^A^	69.44	4.25^D^	-29.16	1.60 ^BC^	**-17.94**	3.65^A^	194.35
	±0.07		± 0.02		± 0.12		± 0.04		± 0.78		± 0.23		± 0.46		± 0.09		± 1.35	
L.S.D _0.05_	0.258	--	0.159	--	0.9764	--	0.3432	--	1.083	--	0.6028	--	1.1398	--	0.3914	--	2.3505	--

#### 3.5.2. Effect on soil electric conductivity (EC) and soluble ions.

Table 6 displays the impact of different treatments on soil EC. All applications, except for T13, indicate an increase in EC when compared to the control treatment. Following rice harvest, the application of azolla compost to the soil resulted in a greater increase in height. Azolla application decreased soil EC and pH, supplied nitrogen, and encouraged rice growth up to a specific salinity level [94]. Soluble K^+^, Ca^2+^, Mg^2+^, Cl^-^, HCO_3_^-^ and So_4_^2 –^ increased with *A. pinnata* and azolla compost application. On the other hand, Soluble Na^+^ decreased with all soil application.

#### 3.5.3. Effects on soil CEC, OM and C/ N ratio in soil after rice harvesting.

According to Table 7, the treatment with *A. pinnata* and azolla compost considerably raised CEC, while OM in the post-harvest soil increased significantly with all soil applications. With 50.408 c mol kg^-1^, the maximum CEC was obtained from the application of the highest rate of azolla compost with 100% NPK. Applying 5 and 10 Mg ha^-1^, on the other hand, raised CEC by 7.07% and 10.67%, respectively. Up to 90% of the soil's adsorbing power comes from soil organic matter, which also promotes granulation, boosts CEC, and releases cations including Ca^+2^, Mg^+2^ and K^+^ during decomposition [95]. The application of 5 and 10 Mg ha^-1^ raised the percentage of OM by 9.84% and 20.56%, respectively, according to the data in Table 8. This could occur as a result of the high temperatures in arid and semi-arid locations, which allow the organic matter that is generated from the breakdown of algae and azolla to oxidize quickly [96,97]. Consecutive azolla cropping with rice plants resulted in a notable increase in the soil's organic carbon content [98]. This, in turn, improved soil fertility by boosting the biomass and proliferation of soil microorganisms. Azolla compost application with highly rate with completely recommendation of NPK fertilizer increased % OM by 33.15%.

**Table 7:** Effects of different treatments on CEC, OM and C/ N ratio of soil after rice harvesting and their relative change.

Treatments	CEC c mol kg^-1^		OM %		C/ N ratio	
	Mean ±Std Error	Relative Change (%)	Mean ±Std Error	Relative Change (%)	Mean ±Std Error	Relative Change (%)
T1	43.997^F^	----	1.493^D^	----	8.718^AB^	----
	± 0.54		± 0.09		± 0.52	
T2	44.511^EF^	**1.16**	1.499^D^	**0.4**	7.599^B^	-12.83
	± 0.60		± 0.08		± 0.45	
T3	47.11^BCD^	**7.07**	1.64^ABCD^	**9.84**	7.912^AB^	**-9.24**
	± 0.36		± 0.03		± 0.14	
T4	48.694^AB^	**10.67**	1.800^ABC^	**20.56**	8.496^AB^	-2.54
	± 0.91		± 0.07		± 0.36	
T5	48.030^BC^	**9.16**	1.538^CD^	**3.01**	7.720^B^	-11.44
	± 0.74		± 0.02		± 0.10	
T6	46.744^CD^	**6.24**	1.67^ABCD^	**11.85**	8.288^AB^	-4.93
	± 0.46		± 0.20		± 0.97	
T7	50.408^A^	**14.57**	1.988 ^A^	**33.15**	9.355^A^	7.3
	± 0.98		± 0.06		± 0.24	
T8	45.42^EFD^	**3.23**	1.525^CD^	**2.14**	8.243^AB^	-5.44
	± 0.36		± 0.15		± 0.79	
T9	46.674^CD^	**6.08**	1.557^BCD^	**4.28**	8.260^AB^	-5.25
	± 1.00		± 0.15		± 0.73	
T10	45.72^EFD^	**3.91**	1.596^BCD^	**6.89**	8.912^AB^	2.22
	± 0.20		± 0.20		± 0.10	
T11	46.140^DE^	**4.87**	1.609^BCD^	**7.76**	8.857^AB^	1.59
	± 0.40		± 0.07		± 0.37	
T12	46.641^CD^	**6.01**	1.829^AB^	**22.5**	9.032^AB^	3.6
	± 0.77		± 0.13		± 0.66	
T13	50.053^A^	**13.76**	1.73^ABCD^	**15.87**	8.032^AB^	-7.86
	± 0.46		± 0.05		± 0.24	
L.S.D _0.05_	1.8558	--	0.2898	--	1.4738	--

T1 → Control (without any applications), T2 → NPK fertilizer (Recommended), T3 → NPK + 5 Mg ha^-1^ *A. pinnata* azolla, T4 → NPK + 10 Mg ha^-1^ *A. pinnata*, T5 → NPK + 7 Mg ha^-1^ azolla compost, T6 → NPK + 10 Mg ha^-1^ azolla compost, T7 → NPK + 14 Mg ha^-1^ azolla compost,T8 → 50%N + PK + 5 Mg ha^-1^ *A. pinnata*, T9 → 50%N + PK + 10 Mg ha^-1^ *A. pinnata*, T10 → 50%N + PK + 7 Mg ha^-1^ azolla compost, T11 → 50%N + PK + 10 Mg ha^-1^ azolla compost, T12 → 50%N + PK + 14 Mg ha^-1^ azolla compost, T13 → NPK + 5 Mg ha^-1^ *A. pinnata* +7 Mg ha^-1^ azolla compost.

Means within a Colum followed by the same letter are not statistically different by LSD at 0.05 level.

**Table 8:** Effects of different treatments on soil available and total NPK of soil after rice harvesting and their relative change.

Treatments	Available NPK						Total NPK					
	N mg Kg^-1^		P mg Kg^-1^		K mg Kg^-1^		N g Kg^-1^		P g Kg^-1^		K g Kg^-1^	
	Mean ±Std Error	Relative Change (%)	Mean ±Std Error	Relative Change (%)	Mean ±Std Error	Relative Change (%)	Mean ±Std Error	Relative Change (%)	Mean ±Std Error	Relative Change (%)	Mean ±Std Error	Relative Change (%)
T1	41.43^H^	----	7.93^H^	----	220.07^G^	----	0.993^I^	----	1.229^H^	----	2.674^H^	----
	± 0.42		± 0.23		± 2.94		± 0.007		± 0.011		± 0.014	
T2	49.66^E^	19.86	9.18^DE^	15.76	268.74^BCD^	22.11	1.145^E^	15.3	1.308^E^	6.42	2.819^E^	5.42
	± 0.42		± 0.16		± 3.59		± 0.010		± 0.013		± 0.010	
T3	52.76^C^	27.34	9.77^BC^	23.2	274.34^BC^	24.66	1.202^C^	21.04	1.361^CD^	10.74	2.868^CD^	7.25
	± 0.20		± 0.08		± 3.67		± 0.004		± 0.007		± 0.012	
T4	54.49^B^	31.52	10.08^B^	27.11	279.04^AB^	26.79	1.229^B^	23.76	1.396^B^	13.58	2.905^B^	8.63
	± 0.44		± 0.12		± 3.73		± 0.009		± 0.011		± 0.009	
T5	50.05^E^	20.8	9.25^D^	16.64	265.34^CD^	20.57	1.155^E^	16.31	1.277^F^	3.9	2.760^F^	3.21
	± 0.22		± 0.14		± 3.55		± 0.005		± 0.010		± 0.011	
T6	52.68^C^	27.15	9.79^BC^	23.45	280.3^BCD^	27.36	1.189^CD^	19.73	1.363^CD^	10.9	2.867^CD^	7.21
	± 0.28		± 0.10		± 3.64		± 0.002		± 0.001		± 0.009	
T7	53.86^B^	30	10.09^B^	27.23	276.43^ABC^	25.61	1.232^B^	24.06	1.387^BC^	12.85	2.887^BC^	7.96
	± 0.40		± 0.13		± 3.69		± 0.008		±0.011		± 0.014	
T8	46.33^F^	11.82	8.59^FG^	8.32	249.53^E^	13.38	1.072^G^	7.95	1.284^EF^	4.47	2.744^FG^	2.61
	± 0.26		± 0.05		± 4.16		± 0.004		±0.003		± 0.007	
T9	46.56^F^	12.38	8.83^EF^	11.34	261.31^D^	18.73	1.092^F^	9.96	1.306^E^	6.26	2.765^F^	3.4
	± 0.40		± 0.19		± 3.49		± 0.006		±0.015		± 0.010	
T10	44.82^G^	8.18	8.29^GH^	4.53	232.57^F^	5.68	1.038^H^	4.53	1.249^GH^	1.62	2.732^FG^	2.16
	± 0.36		± 0.1		± 7.48		± 0.007		±0.007		±0.009	
T11	45.18^G^	9.05	8.42^G^	6.17	245.74^E^	11.66	1.053^H^	6.04	1.262^FG^	2.68	2.712^G^	1.42
	± 0.25		± 0.06		± 3.28		± 0.006		±0.007		± 0.010	
T12	51.58^D^	24.49	9.72^C^	22.57	270.83^BCD^	23.06	1.175^D^	18.32	1.348^D^	9.68	2.846^DE^	6.43
	± 0.49		± 0.08		± 3.62		± 0.007		±0.007		± 0.012	
T13	56.71^A^	36.88	10.59^A^	33.54	286.45 ^A^	30.16	1.25^A^	25.88	1.427^A^	16.11	2.970^A^	11.06
	± 0.46		± 0.08		± 4.11		± 0.007		±0.007		± 0.20	
L.S.D _0.05_	1.0509	--	0.3634	--	11.635	--	0.0193	--	0.0273	--	0.0336	--

Regarding how various treatments affected the C/N ratio in soils following rice harvest, Table 8's data indicates that all applications significantly increased the ratio when compared to the control in uncontaminated soil, while some applications significantly decreased it when compared to the control in contaminated soil. The C/N ratio was reduced by 9.24% and 2.54%, respectively, by applying 5 and 10 Mg ha^-1^. Compost can be characterized as mature only when the C/N ratio is below 20. The lower the C:N ratio, the more rapidly nitrogen will be released into the soil for immediate crop use [99,100]. By raising the amount of organic matter in the soil, boosting microbial activity, and increasing CEC, azolla enhances soil properties. In addition to enriching soil Organic carbon, the breakdown of Azolla biomass creates beneficial microbial communities that improve nutrient cycling and soil health in general [101]. Additionally, humic compounds derived from Azolla improve nutrient retention and the availability of essential cations like potassium (K⁺), calcium (Ca²⁺), magnesium (Mg²⁺), and ammonium (NH₄⁺) by increasing CEC [102].

### 3.6. Effects on available and total NPK in soil after rice harvesting

As indicated in Table 8, all soil treatments considerably raised the post-harvest soil's available and total nitrogen, phosphorus, and potassium levels. The 100%NPK + 5 Mg ha^-1^ *A. pinnata* +7 Mg ha^-1^ azolla compost (T13) combination produced the maximum amount of accessible nitrogen (56.71 mg Kg^-1^). *A. pinnata* and its compost treatments with full NPK fertilizer recommendations had higher available and total NPK than either *A. pinnata* or azolla compost treatments alone. This increase in available and total NPK was statistically significant at the 0.05 level of probability. *A. pinnata* application at rates of 5 and 10 Mg ha^-1^ resulted in increases in available N of 27.34% and 31.52%, respectively. Applying azolla compost at a high rate while fully recommending NPK fertilizer improved the amount of available nitrogen by 30.00%. On the other hand, total N rose by 21.04% and 23.76%, respectively, with applications of 5 and 10 Mg ha^-1^. Total N rose by 24.06% when azolla compost was applied at a high rate and NPK fertilizer was fully recommended. Azolla increases soil fertility by increasing organic carbon, organic matter, total nitrogen, available NPK, and water-holding capacity. Furthermore, it improves soil texture [103–108]. The chemical analysis indicates that the azolla used in our study has a high N content (Table 2). It is likely that the increase of total N is related to the fact that N content supplied by 10 Mg ha^-1^ of *A. pinnata* was higher than N supplied by 5 Mg ha^-1^ of *A. pinnata*.

Concerning the effects of different treatments on available and total P in soils after rice harvesting, the data in Table 8 show a significant increase with all applications compared to the control. High available P was obtained with the treatment 13 compared to other treatments, at 10.59 mg Kg^-1^. *A. pinnata* at rate 5 and 10 Mg ha^1^ increased available P by 23.20%, and 27.11%, respectively. Azolla compost application at a high rate with the full recommendation of NPK fertilizer increased available P by 27.23%. Whereas application of azolla at rates of 5 and 10 Mg ha^-1^ increased total P by 10.74% and 13.58%, respectively. Azolla compost application at a high rate with completely recommendation of NPK fertilizer increased total P by 12.85%. Setiawati et al., [80] indicated that the higher significant mean values of small phosphorous in the soil were due to high phosphorous content of azolla, it may also be as a result of the decrease of soil pH values. Available phosphorus could be increased in soil because of the excretions of organic acids by biofertilizer inoculation which convert slight soluble CO_3_ (PO_4_)_2_ to soluble di-and monobasic phosphates [109]. Singh et al., [110] reported that that incorporation of azolla into rice fields also increase P availability in soils, as the decomposition of azolla’s biomass may lead to reduction and chelation. Increased P availability on the incorporation of azolla ultimately increase the uptake of P by rice plants and their P concentrations [104].

Results in Table 8 indicated significant increment of available and total K, the highest available K was recorded in T13 with (286.45 mg Kg^-1^), whereas, the application of 5 and 10 Mg ha^1^ increased available K by 24.66%, and 26.79%, respectively. Azolla compost application with highly rate with completely recommendation of NPK fertilizer increased available K by 25.61%. Whereas application of azolla at rate of 5 and 10 Mg ha^-1^ increased total K by 7.25% and 8.63%, respectively. Azolla compost application at a high rate with completely recommendation of NPK fertilizer increased total K by 7.96%. The significantly high mean values of K may be due to high mean values of nitrogen which enhanced its uptake.

Sharma [111] reported that soil pH, organic carbon and available N, P and K increased with increasing azolla application. NPK elements could be increased with pH reduction in root zone by biofertilizer inoculations. Potassium in soil could be released from soil (clay) minerals due to organic acids through hydrolysis or solution processes caused by organic acids [109].

### 3.7. Effects on available and total Zn, Cu and Pb in soil after rice harvesting

Available and total Zinc, Copper and Lead in the post-harvest soil decreased significantly with all soil applications compared to control as shown in Table 9. Application of 5 and 10 Mg ha^-1^ decreased available Zn by 8.42% and 9.28%, respectively. Azolla compost application with highly rate with completely recommendation of NPK fertilizer decreased available Zn by 0.79%. Whereas application of 5 and 10 Mg ha^-1^ decreased total Zn by 12.38% and 13.97%, respectively. Azolla compost application with highly rate with completely recommendation of NPK fertilizer decreased total Zn by 9.29%, respectively.

**Table 9:** Effects of different treatments on soil available and total Zn, Cu and Pb after rice harvesting and their relative change.

Treatments	Total Zn, Cu and Pb						Available Zn, Cu and Pb					
	Pb (mg Kg^-1^)		Cu (mg Kg^-1^)		Zn (mg Kg^-1^)		Pb (mg Kg^-1^)		Cu (mg Kg^-1^)		Zn (mg Kg^-1^)	
	Relative Change (%)	Mean ±Std Error	Relative Change (%)	Mean ±Std Error	Relative Change (%)	Mean ±Std Error	Relative Change (%)	Mean ±Std Error	Relative Change (%)	Mean ±Std Error	Relative Change (%)	Mean ±Std Error
T1	4.287^A^	----	7.876^A^	----	4.065^A^	----	224.64^A^	----	66.088^A^	----	82.293^A^	----
	± 0.010		± 0.027		± 0.030		± 0.456		± 0.005		± 0.108	
					
T2	4.269^A^	-0.41	7.818^AB^	-0.73	4.053^AB^	-1.2	223.87^A^	-0.34	65.757^B^	-0.5	82.289^A^	-0.004
	± 0.010		± 0.027		± 0.030		± 0.556		± 0.005		± 0.233	
T3	3.926^F^	-8.42	7.164^H^	-9.04	3.959^AB^	-10.6	196.82^G^	-12.38	61.868^L^	-6.38	79.669^CD^	-3.18
	± 0.008		± 0.031		± 0.078		± 0.387		± 0.005		± 0.225	
T4	3.889^F^	-9.28	7.141^H^	-9.33	3.895^AB^	-17	193.24^H^	-13.97	61.402^M^	-7.09	79.348^DE^	-3.57
	± 0.014		± 0.028		± 0.080		± 0.380		± 0.004		± 0.224	
T5	4.177^B^	-2.56	7.631^DE^	-3.11	3.999^AB^	-6.6	206.62^D^	-8.02	65.267^C^	-1.24	80.960^B^	-1.61
	± 0.013		± 0.027		± 0.029		± 0.406		± 0.005		± 0.229	
T6	4.248^A^	-0.9	7.607^E^	-3.41	3.983^AB^	-8.2	207.02^D^	-7.84	64.622^D^	-2.21	80.150^C^	-2.6
	± 0.010		± 0.026		± 0.032		± 0.407		± 0.005		± 0.227	
T7	4.253^A^	-0.79	7.497^F^	-4.81	3.928^AB^	-13.7	203.76^E^	-9.29	64.143^E^	-2.94	79.411^D^	-3.5
	± 0.009		± 0.026		± 0.029		± 0.401		± 0.005		± 0.225	
T8	4.043^D^	-5.69	7.689^DE^	-2.37	4.015^AB^	-5	202.68^E^	-9.77	62.888^H^	-4.84	79.989^CD^	-2.79
	± 0.007		± 0.027		± 0.080		± 0.398		± 0.005		± 0.226	
T9	4.018^D^	-6.27	7.665^CDE^	-2.67	4.014^AB^	-5.1	199.42^F^	-11.22	62.448^J^	-5.5	79.842^CD^	-2.97
	± 0.005		± 0.027		± 0.029		± 0.392		± 0.005		± 0.226	
T10	4.174^B^	-2.63	7.799^B^	-0.97	4.039^AB^	-2.6	220.51^B^	-1.83	63.664^F^	-3.66	81.076^B^	-1.47
	± 0.014		± 0.027		± 0.068		± 0.866		± 0.005		± 0.229	
T11	4.161^B^	-2.93	7.722^C^	-1.95	4.038^AB^	-2.7	220.98^B^	-1.62	63.191^G^	-4.38	80.254^C^	-2.47
	± 0.041		± 0.027		± 0.079		± 0.868		± 0.005		± 0.227	
T12	4.107^C^	-4.19	7.690^CD^	-2.36	4.034^AB^	-3.1	216.48^C^	-3.63	62.638^I^	-5.22	80.089^C^	-2.67
	± 0.012		± 0.027		±0.029		± 0.850		± 0.005		± 0.227	
T13	3.926^F^	-8.42	7.323^G^	-7.02	3.890^B^	-17.5	198.88^F^	-11.46	62.162^K^	-5.94	78.697^E^	-4.36
	± 0.010		±0.014		± 0.080		± 0.470		± 0.005		± 0.315	
L.S.D _0.05_	0.0438	--	0.0758	--	0.1709	--	1.6003	--	0.0147	--	0.655	--
Recommended concentration according to [112]	--	--	--	--	--	--	200	--	63	--	70	--

Concerning the effects of different treatments on available and total Cu in soils after rice harvesting, the data in Table 9 show a significant decrease with all applications compared to the control. The lowest available Cu was obtained with the treatment of 10 Mg ha^-1^ *A. pinnata* compared to other treatments, at 9.33% less than the control. Azolla compost application at a high rate with the full recommendation of NPK fertilizer decreased available Cu by 4.81%. Whereas application of 5 and 10 Mg ha^-1^ decreased total Cu by 6.38% and 7.09%, respectively. Azolla compost application at a high rate with the full recommendation of NPK fertilizer decreased total Cu by 2.94%.

The results in Tables 9 indicated significant decrement in available and total Pb. The lowest available Pb was recorded in T13 at 3.89 mg Kg^-1^. The application of 5 and 10 Mg ha^1^ decreased available Pb by 10.60%, and 17.00%, respectively. Azolla compost application at a high rate with the full recommendation of NPK fertilizer decreased available Pb by 13.70%. Furthermore, the application of 5 and 10 Mg ha^-1^ decreased total Pb by 3.18% and 3.57%, respectively. Azolla compost application at a high rate with the full recommendation of NPK fertilizer decreased total Pb by 3.50%.

*A. pinnata* has finer roots than *Azolla macrophylla*, which gives it a larger surface area and a greater capacity to absorb heavy metals [113]. However, *A. macrophylla* has a high biomass and is highly active in the environment, so even though it has a great potential to grow in polluted areas, plants may be exposed to harsh conditions. Additionally, living *A. filiculoides* can be used to purify polluted water because of its capacity to remove Pb+ ^2^, Cd+ ^2^ Ni+ ^2^and Zn+ ^2^ by approximately 61%, 57%, 68%, and 74% [114]. *A. filiculoides* can grow in solutions containing these metal ions with initial concentrations of 4 mg/L within 15 days.

## 4. Conclusion

The results revealed that the integration of *A. pinnata* and azolla compost had significant positive effects:it inhibited the pH increasing, and led to the increase of CEC, %OM, available and total NPK, and enhanced the grain yield of rice. *A. pinnata* proved to be the most successful in decreasing the toxicity effect of the studied heavy metals and improving fertility (available and total NPK) of the soil, resulting in a greater increase in rice yield compared to azolla compost application alone. Consequently, the best results were recorded with the interaction between *A. pinnata* and azolla compost in soils at a rate of 100% NPK + 5 Mg ha^-1^ *A. pinnata* +7 Mg ha^-1^ azolla compost especially for the reduction of available and total Pb concentration in contaminated soil. The total Pb concentration remained high, although overall zinc and copper levels in our study dropped to a safe level, allowing for the use of treatments in accordance with Canadian soil quality guidelines.
